# Supplementary figures and images for: Multimodal mapping of cell types and projections in the central nucleus of the amygdala
Source: eLife. 2023 Jan 20;12:e84262. doi: 10.7554/eLife.84262 (PMC9977318; doi:10.7554/eLife.84262)

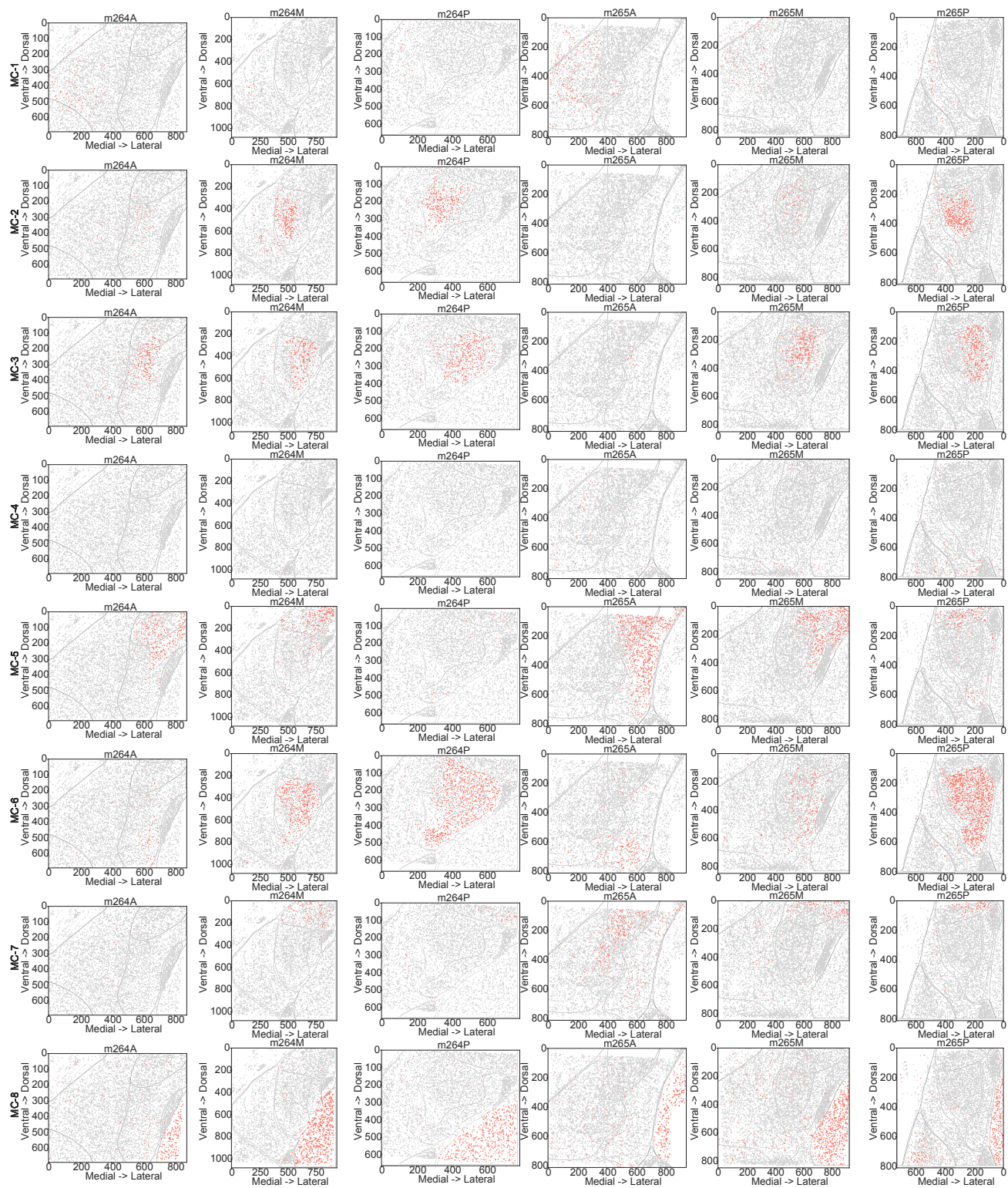

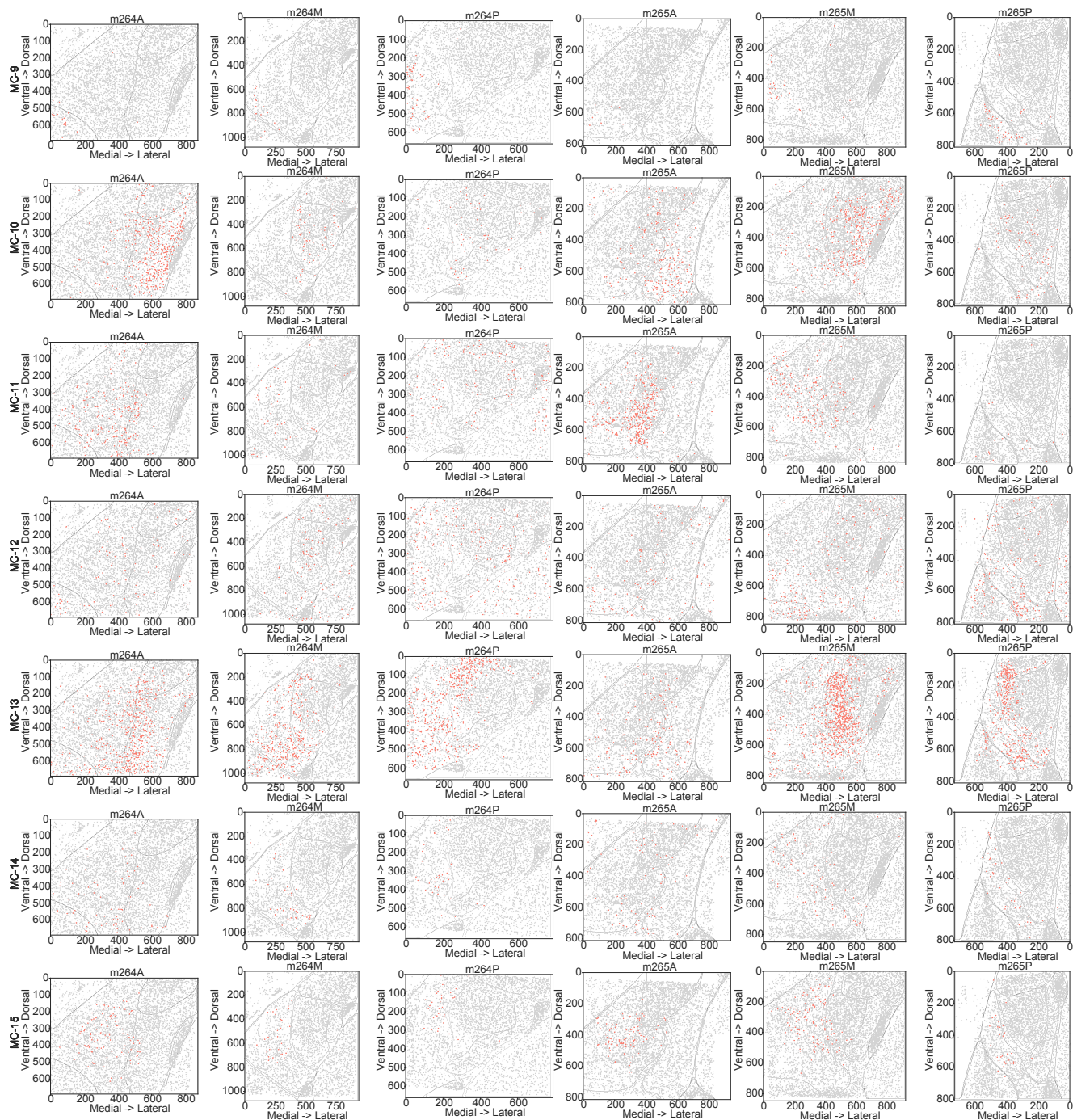

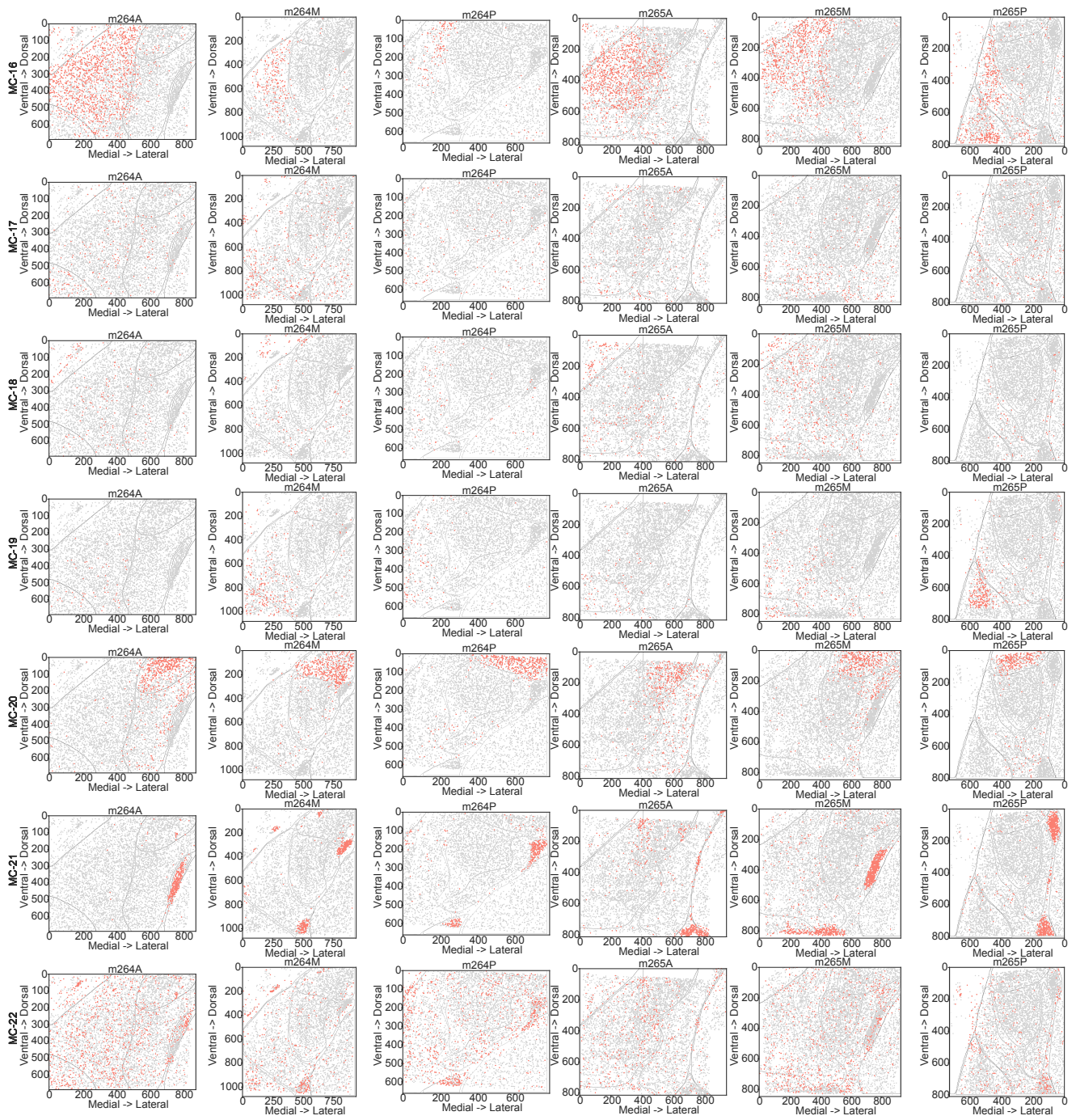

Supplement: Figure 4—source data 1. — Each dot indicates the centroid position of a neuron. Each column is a sample from selected animal. Panels are maximum axial projections of the entire volumes. [file elife-84262-fig4-data1.pdf]

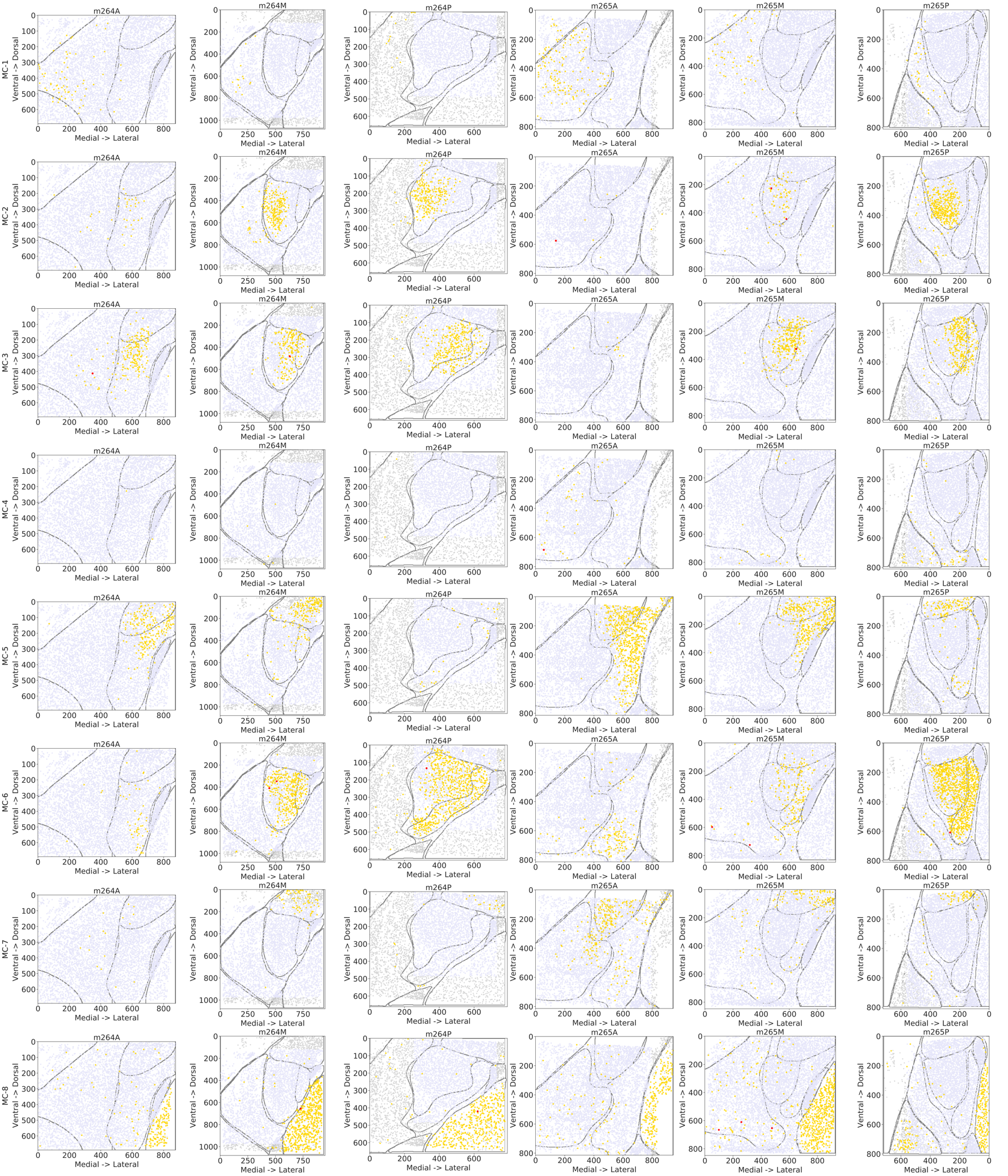

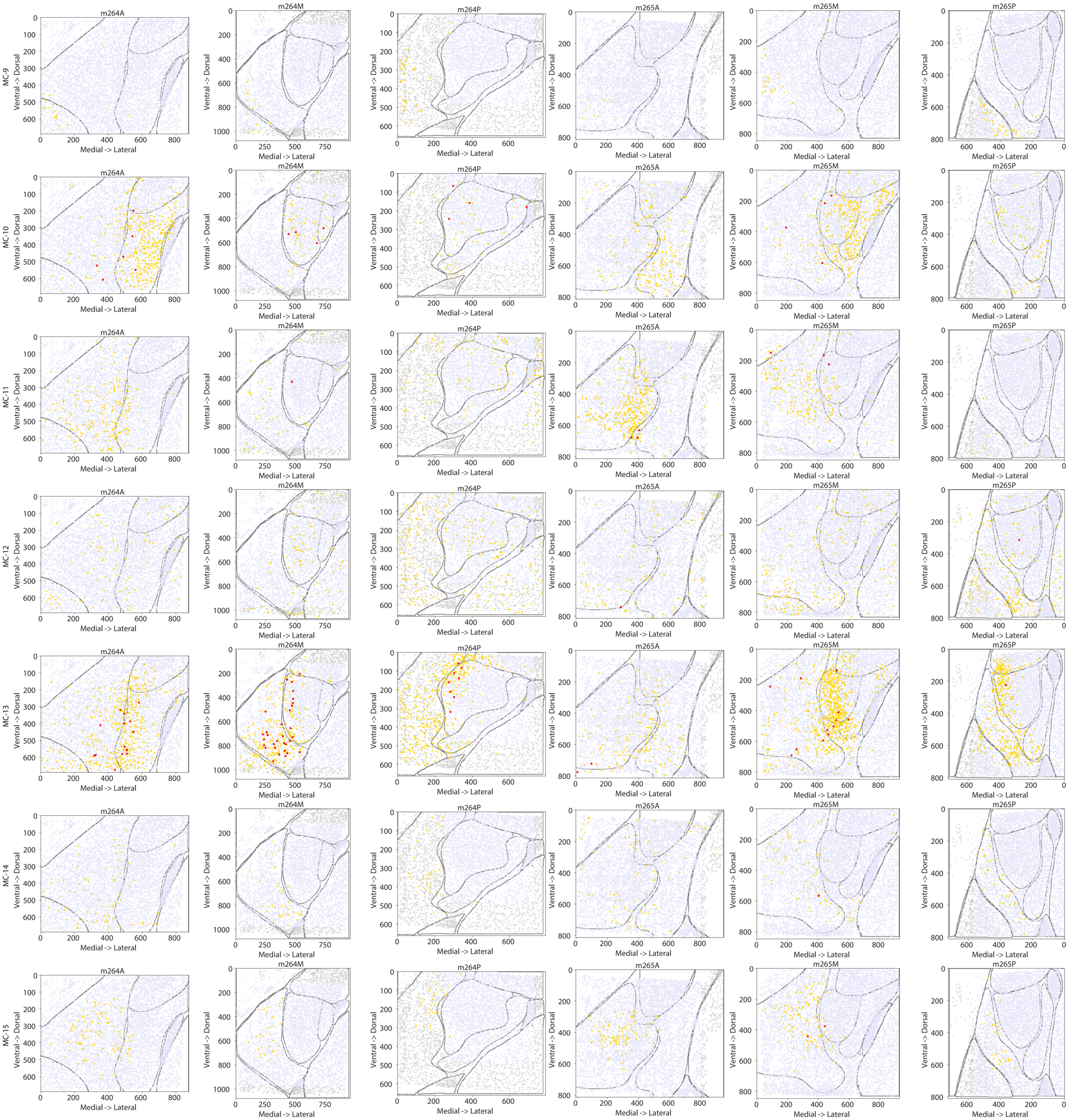

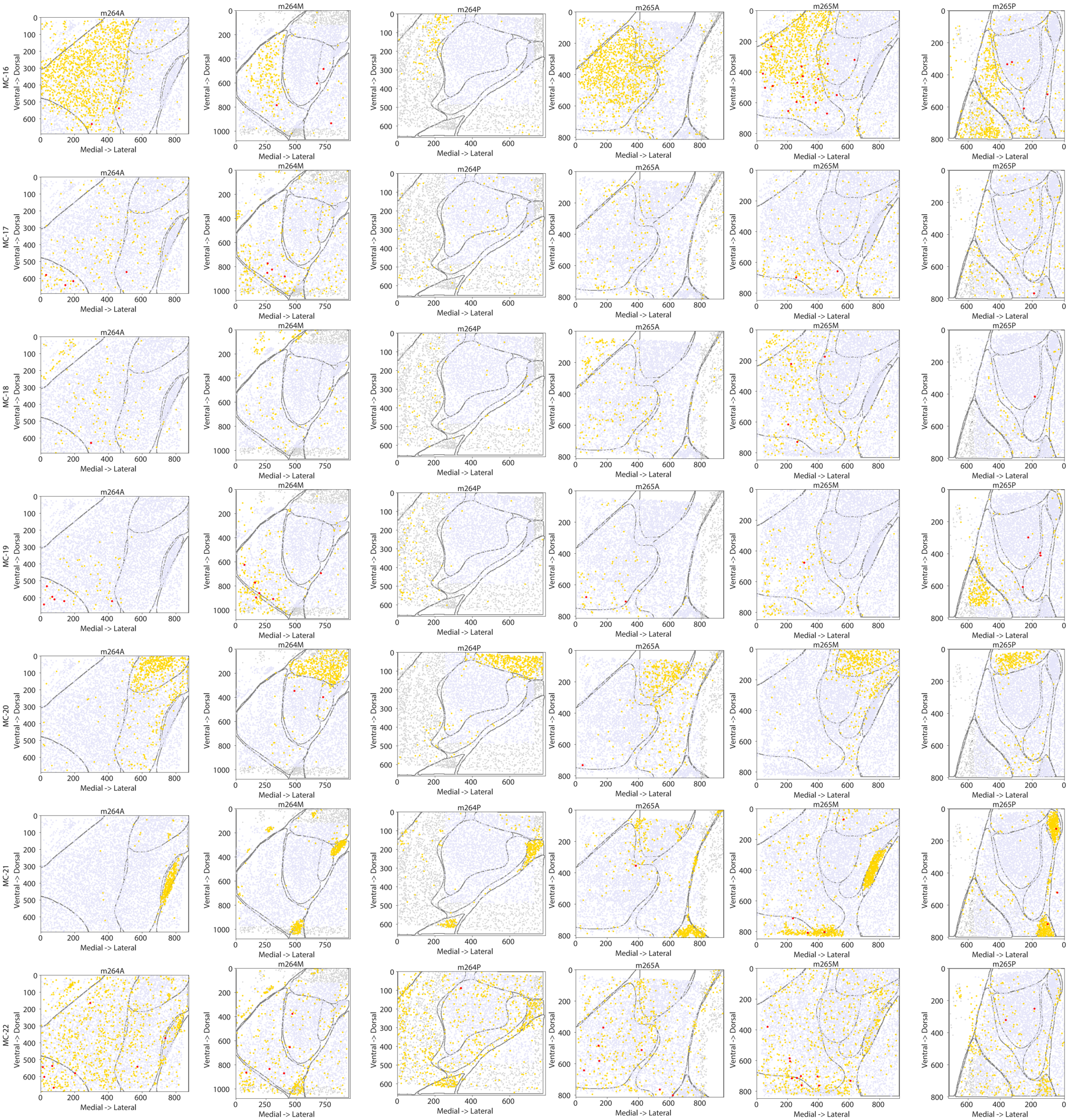

Supplement: Figure 5—source data 1. — Each dot indicates the centroid position of a neuron. Each column is a sample from a selected animal. Panels are maximum axial projections of the entire volumes. [file elife-84262-fig5-data1.pdf]

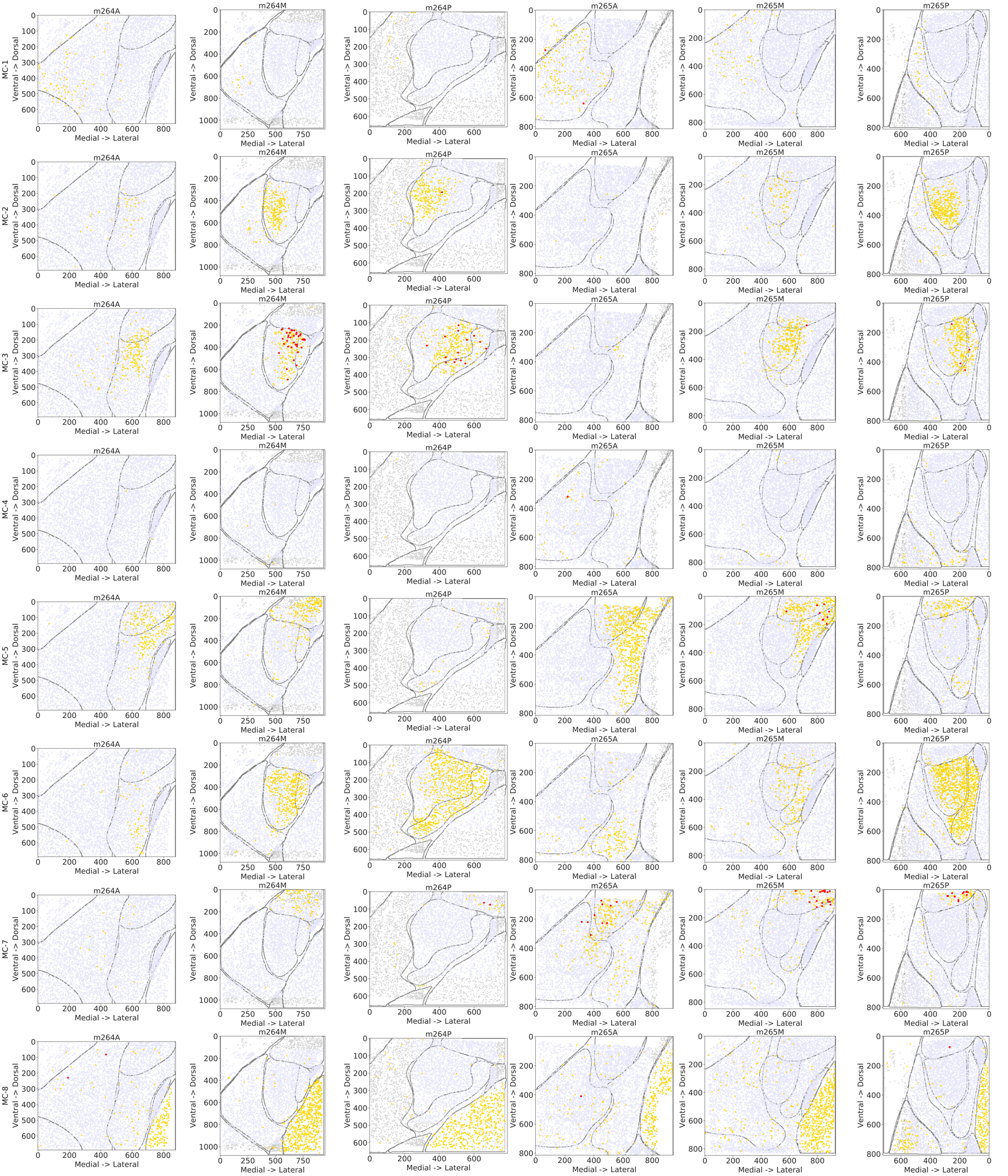

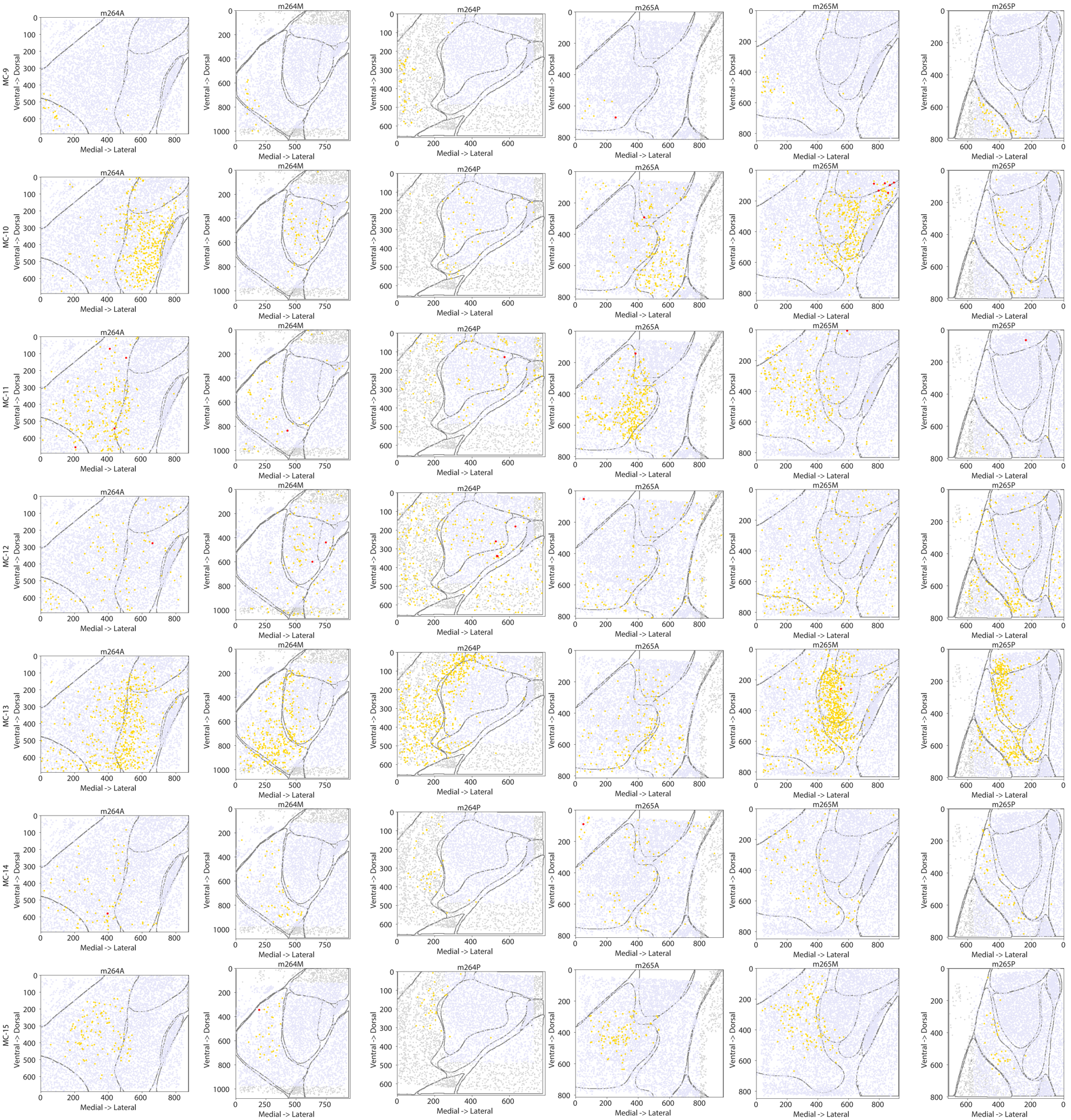

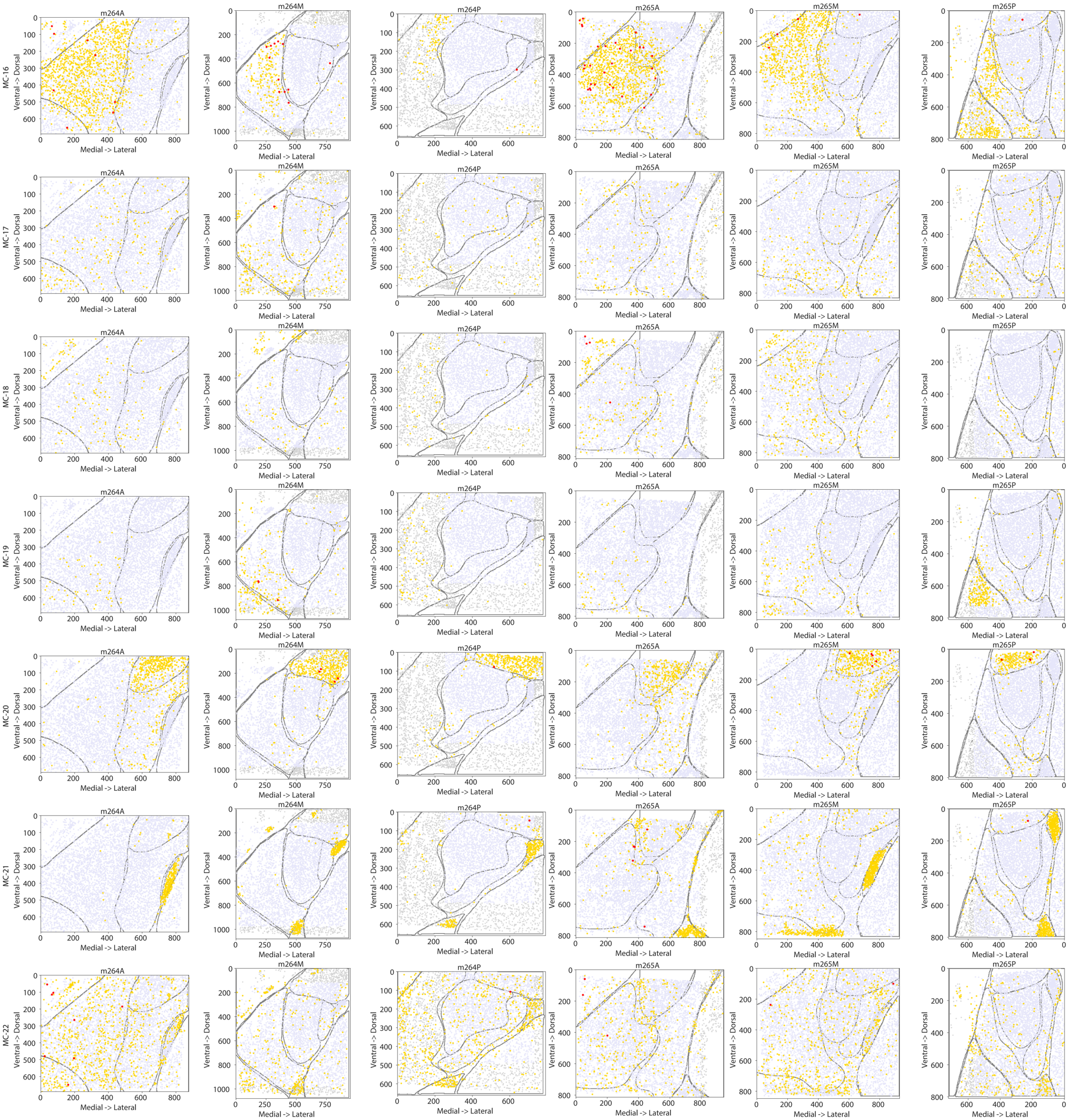

Supplement: Figure 5—source data 2. — Each dot indicates the centroid position of a neuron. Each column is a sample from a selected animal. Panels are maximum axial projections of the entire volumes. [file elife-84262-fig5-data2.pdf]

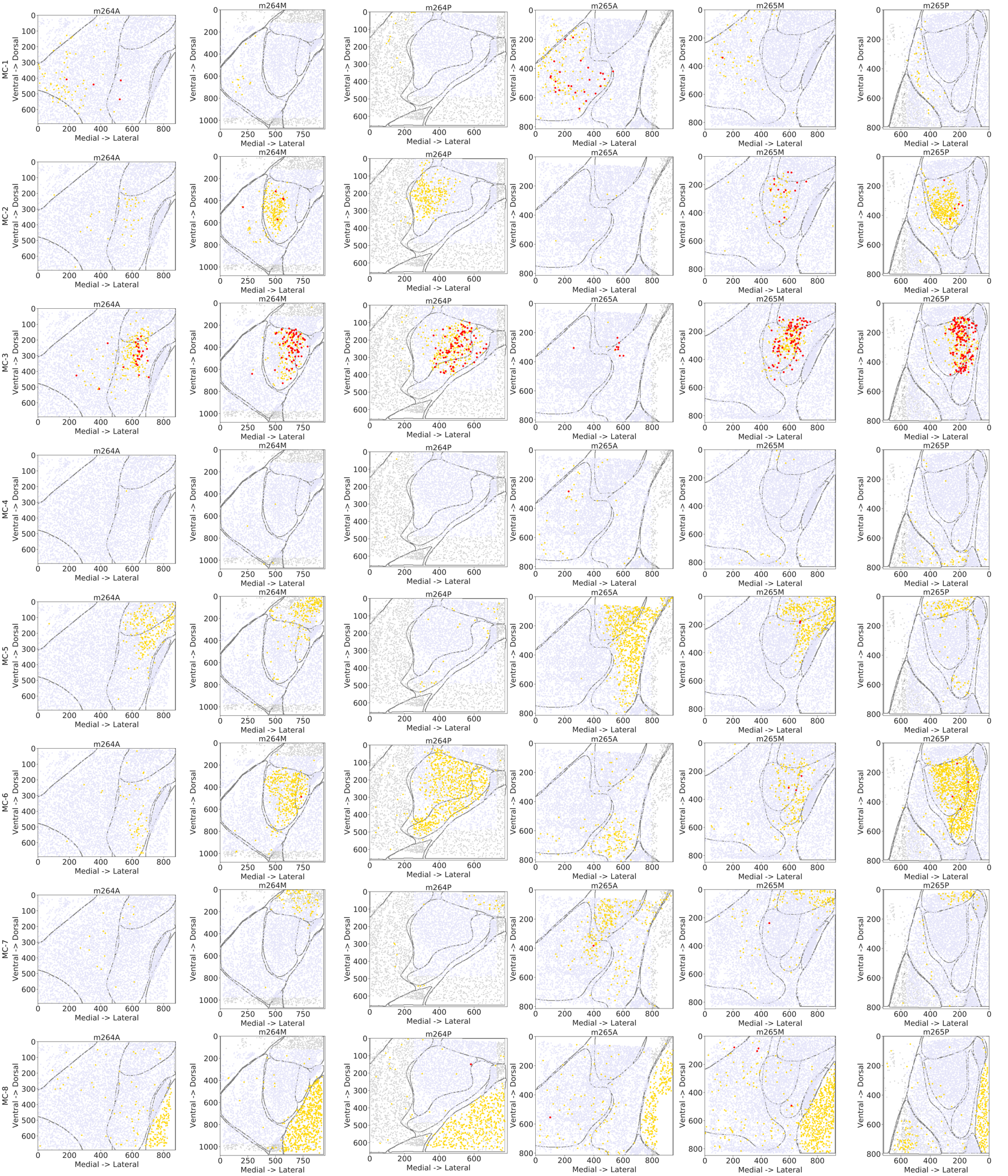

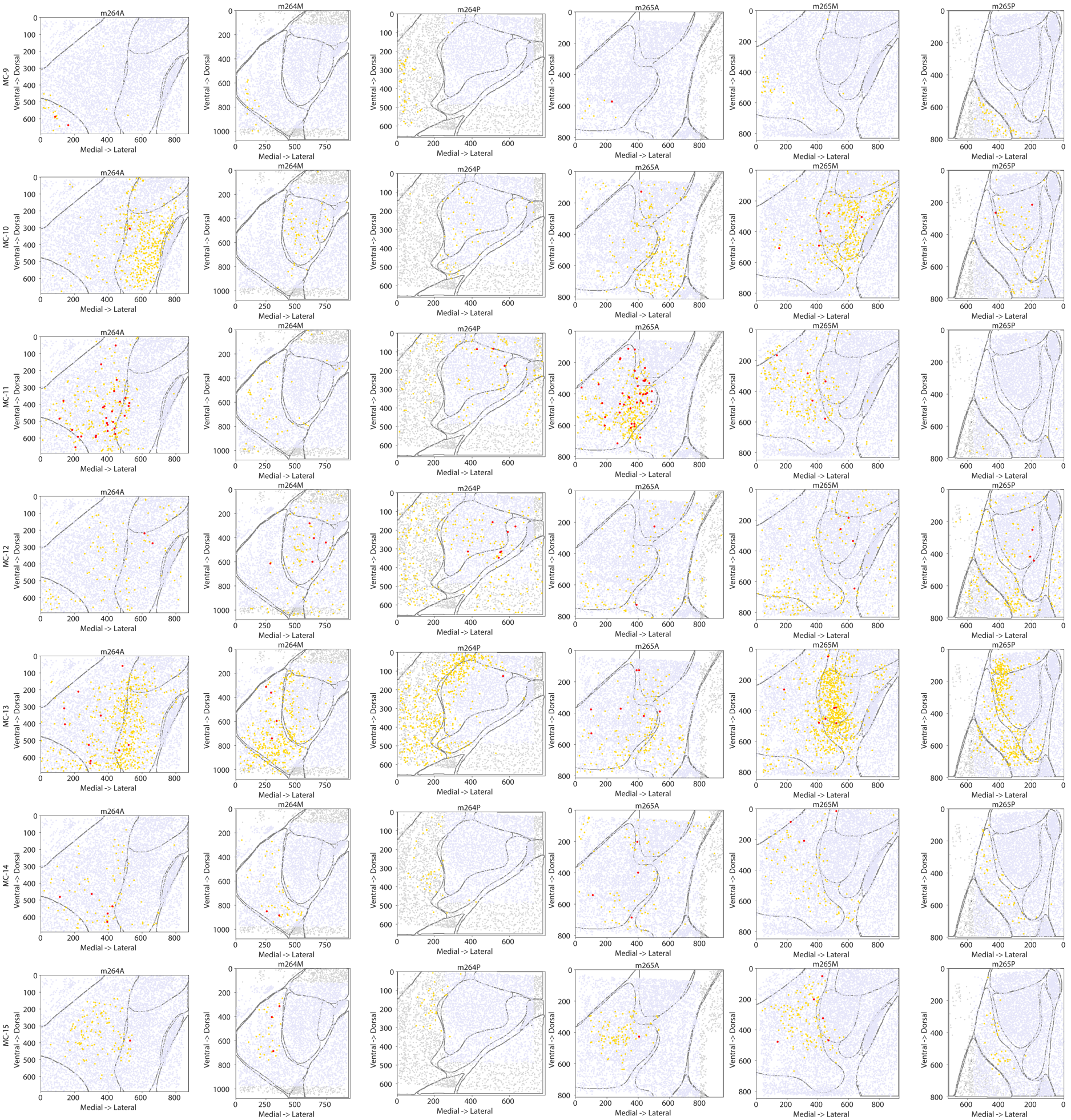

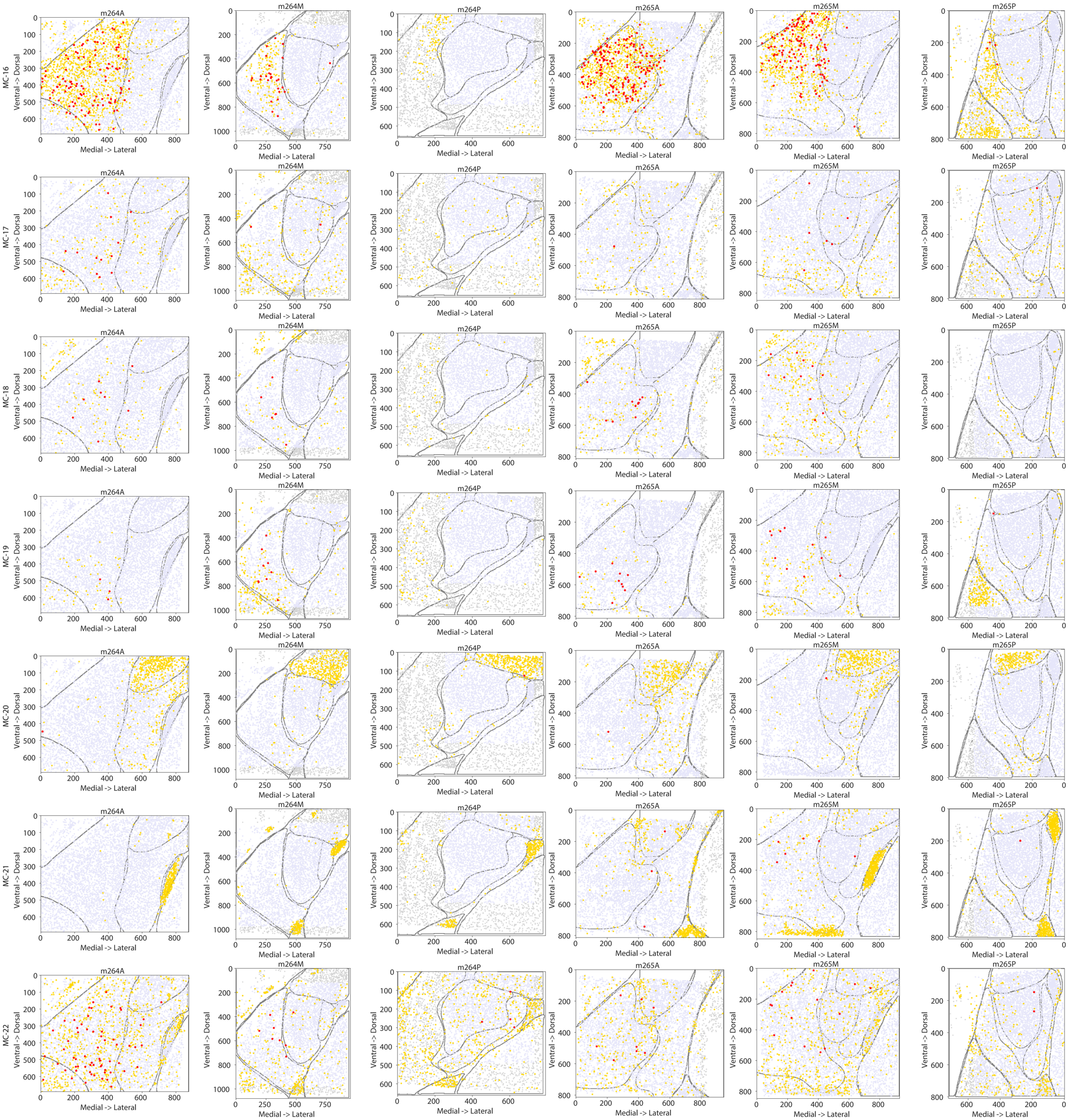

Supplement: Figure 5—source data 3. — Each dot indicates the centroid position of a neuron. Each column is a sample from a selected animal. Panels are maximum axial projections of the entire volumes. [file elife-84262-fig5-data3.pdf]

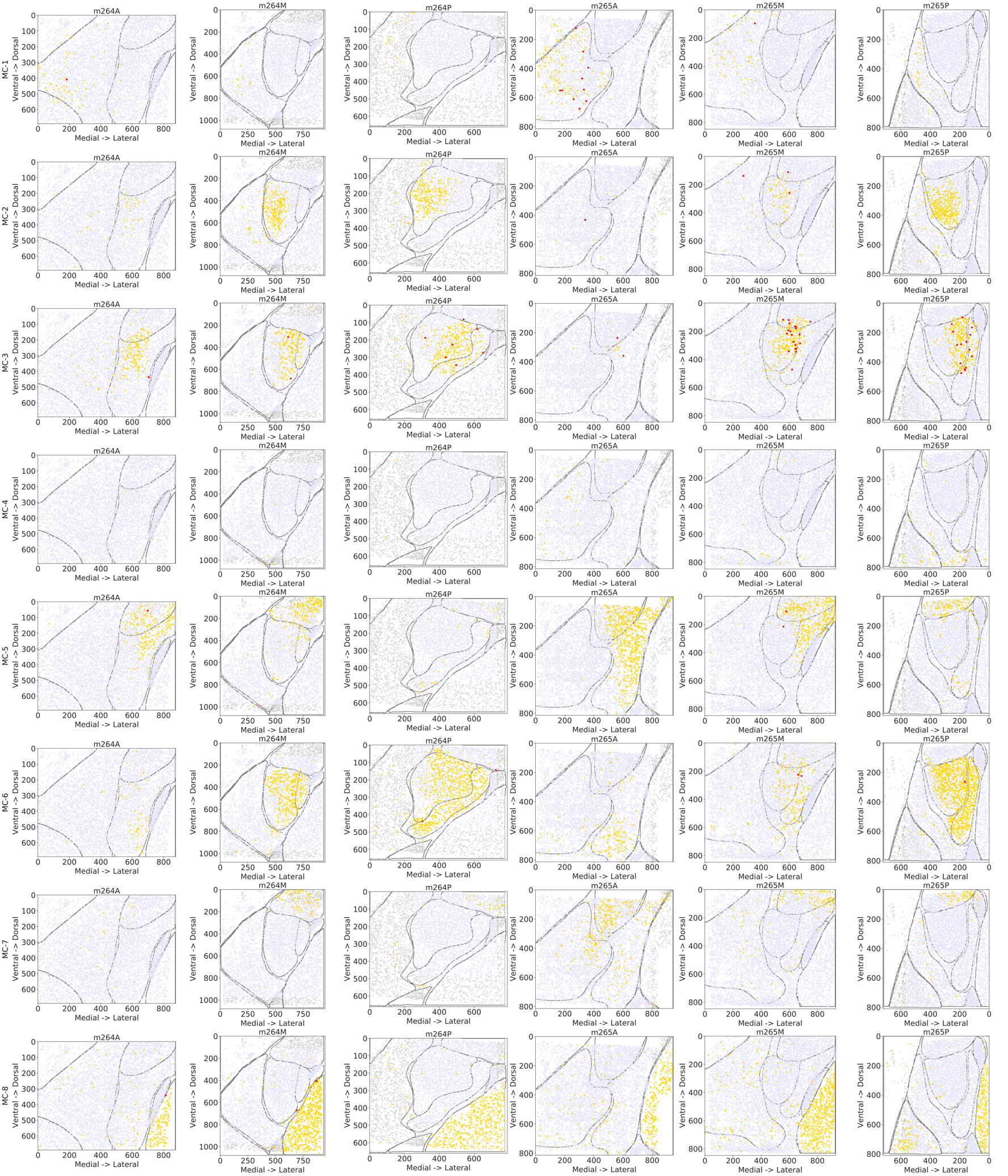

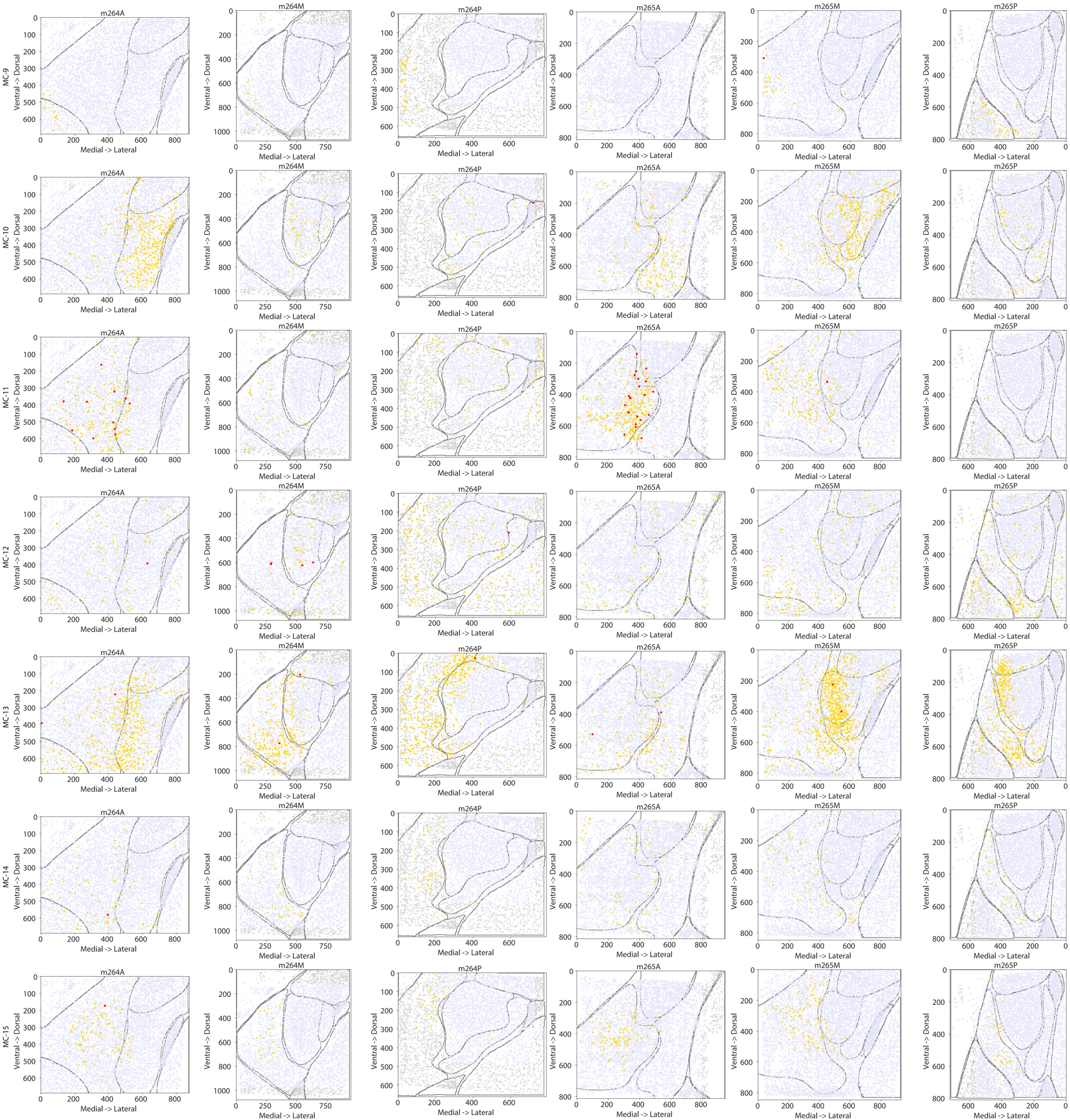

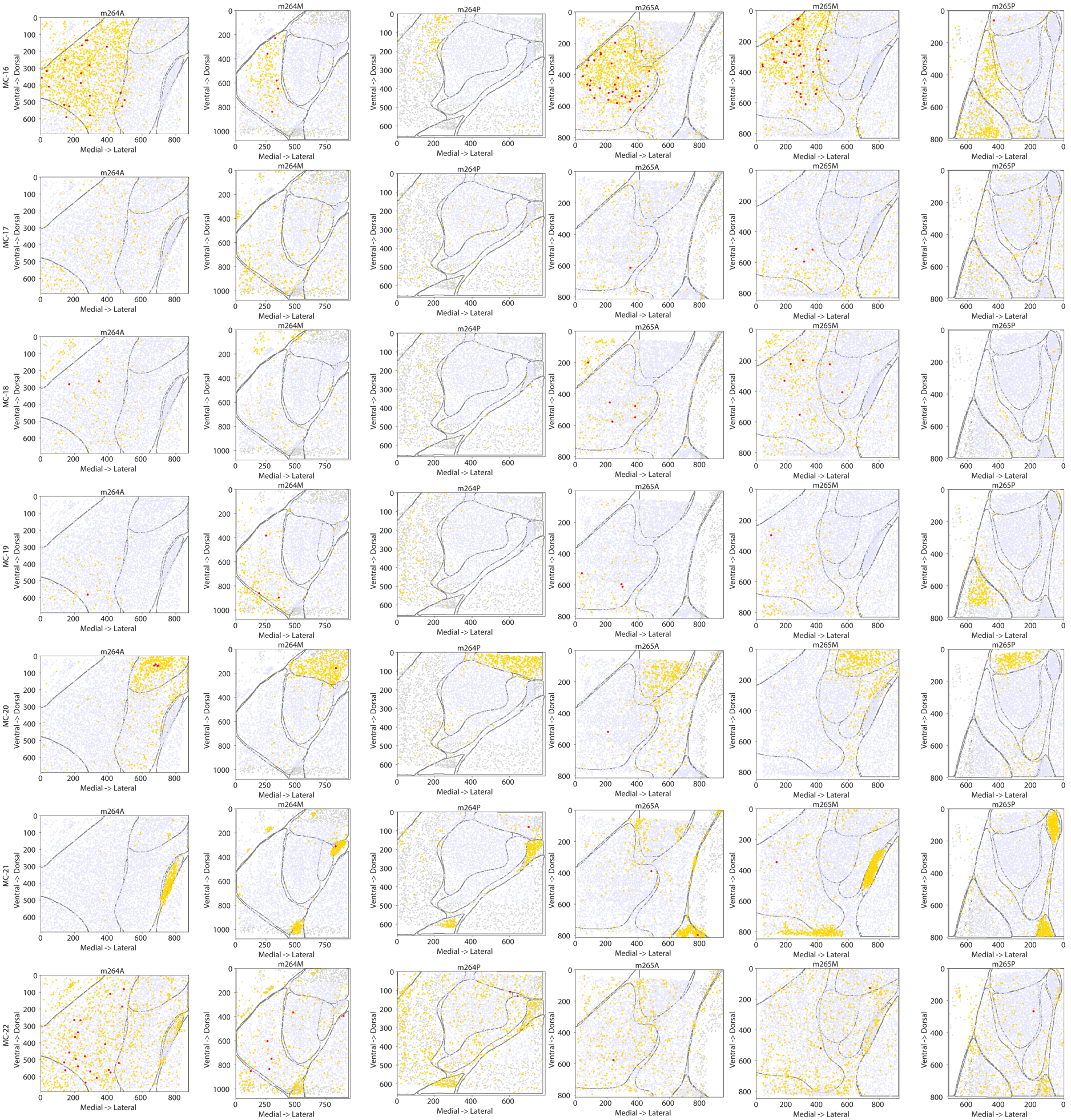

Supplement: Figure 5—source data 4. — Each dot indicates the centroid position of a neuron. Each column is a sample from a selected animal. Panels are maximum axial projections of the entire vol umes. [file elife-84262-fig5-data4.pdf]

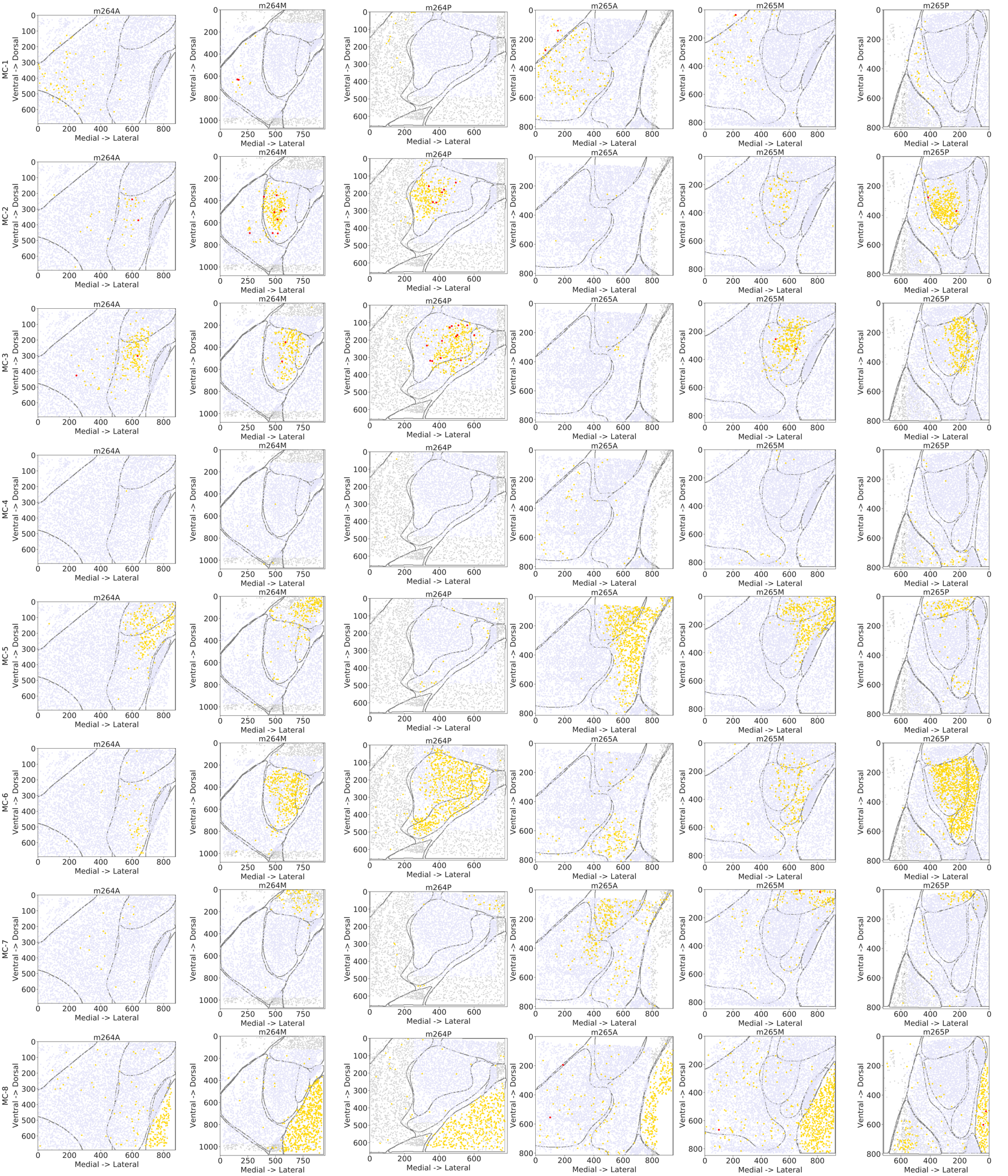

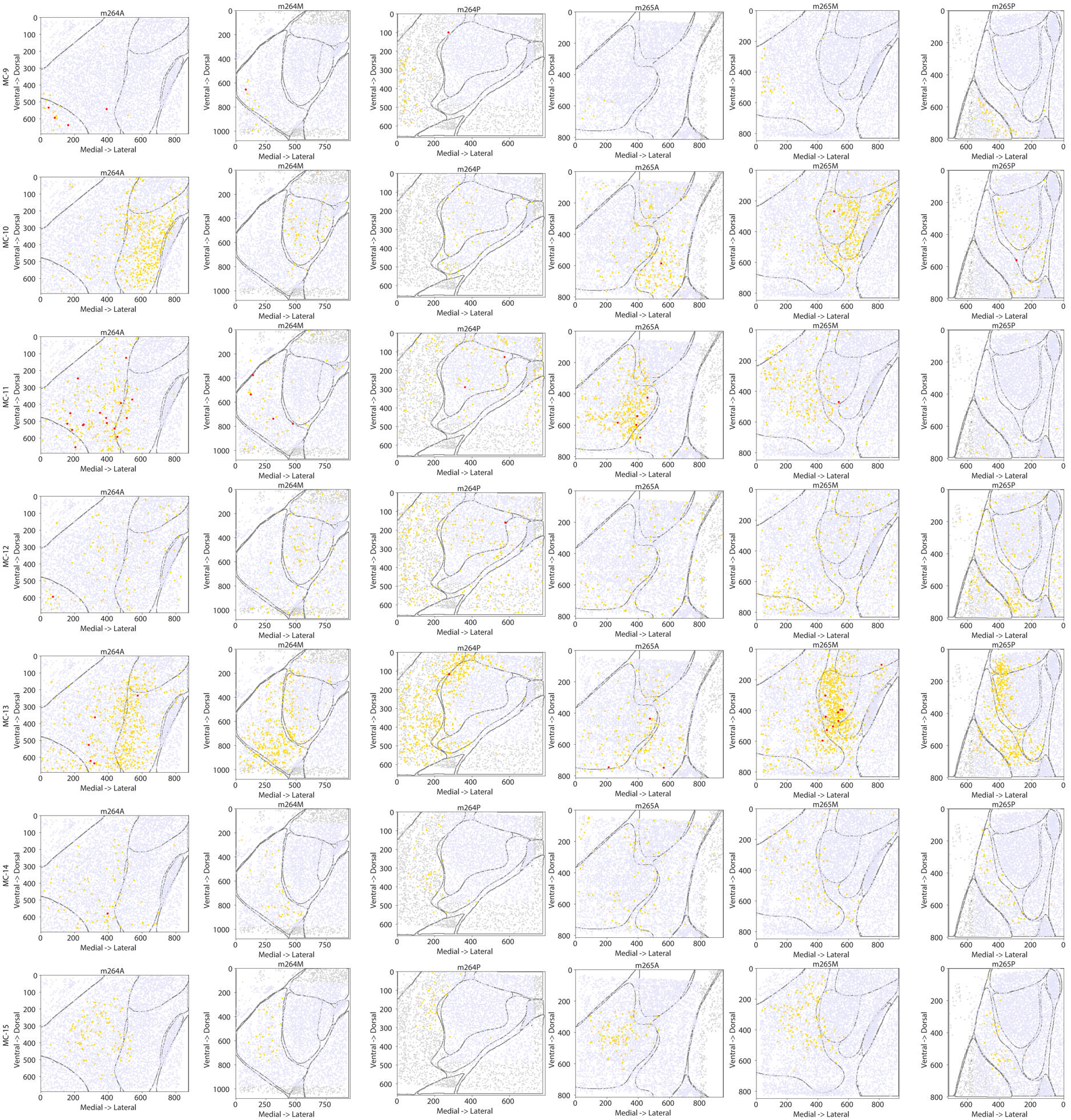

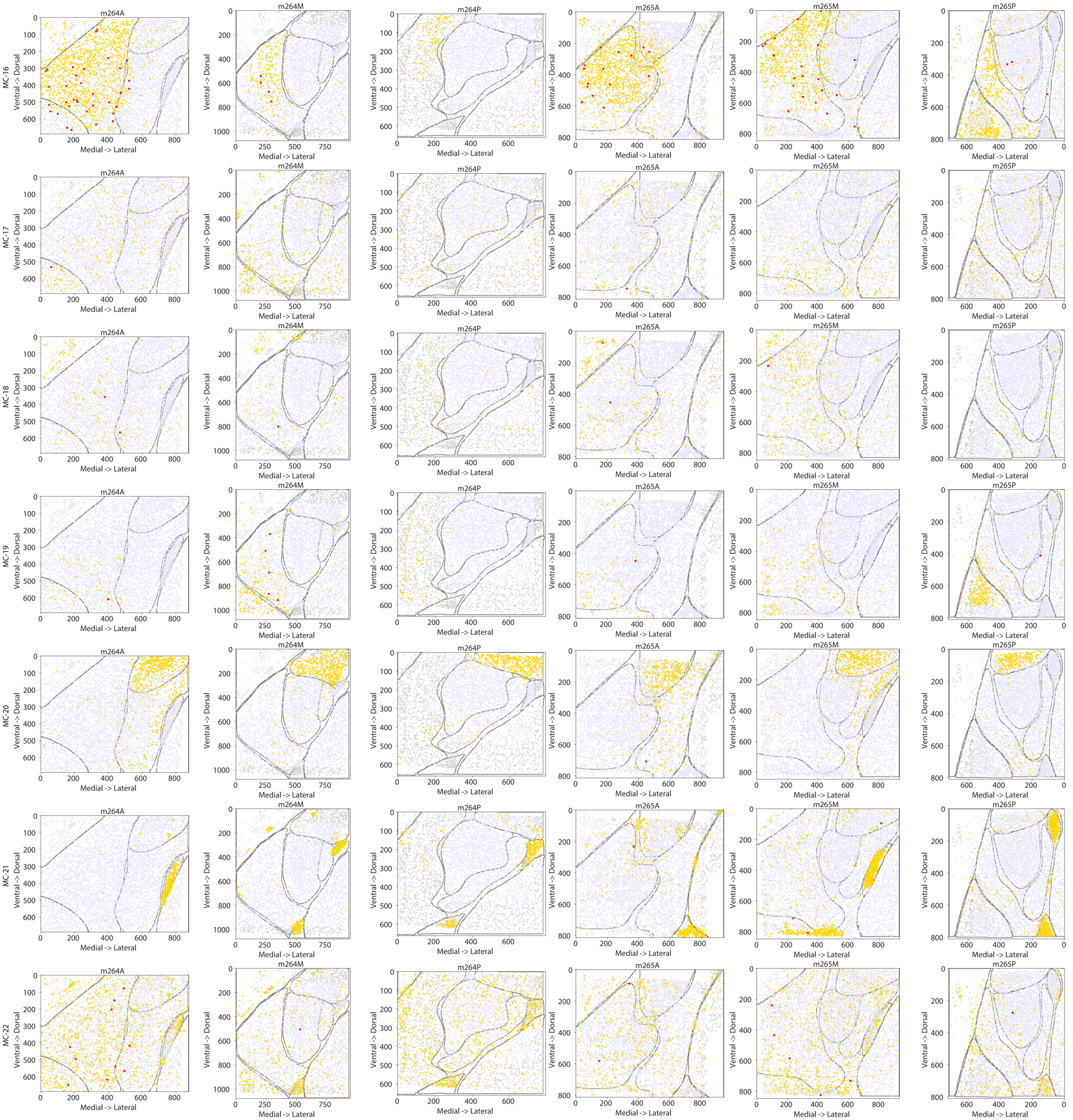

Supplement: Figure 5—source data 5. — Each dot indicates the centroid position of a neuron. Each column is a sample from a selected animal. Panels are maximum axial projections of the entire volumes. [file elife-84262-fig5-data5.pdf]
