# Supplementary material for: Multimodal mapping of cell types and projections in the central nucleus of the amygdala
Source: eLife. 2023 Jan 20;12:e84262. doi: 10.7554/eLife.84262 (PMC9977318; doi:10.7554/eLife.84262)
Supplement: Supplementary file 2. [file elife-84262-supp2.docx]

**Supplementary File 2.** Marker-genes used for EASI-FISH experiment in the CEA

|  | **Genes** | **HCR hairpin** | **Fluorophores** |
| --- | --- | --- | --- |
| Round 1 | *Oprk1* | B1 | AF-488 |
|  | *Scn4b* | B3 | AF-546 |
|  | *Vipr2* | B5 | JF-699 |
| Round 2 | *Npy1r* | B1 | AF-488 |
|  | *Sema3c* | B3 | AF-546 |
|  | *Drd1* | B5 | JF-699 |
| Round 3 | *Drd2* | B1 | AF-488 |
|  | *Htr1b* | B3 | AF-546 |
|  | *Htr2c* | B5 | JF-699 |
| Round 4* | *Ebf1* | B1 | AF-488 |
|  | *Crym* | B3 | AF-546 |
|  | *-* | - | - |
| Round 5* | *Cyp26b1* | B5 | AF-488 |
|  | *Crh* | B1 | AF-546 |
|  | *-* | - | - |
| Round 6 | *Dlk1* | B1 | AF-488 |
|  | *Vdr* | B3 | AF-546 |
|  | *Gpx3* | B5 | JF-699 |
| Round 7 | *Nefm* | B1 | AF-488 |
|  | *Vgf* | B3 | AF-546 |
|  | *Ppp1r1b* | B5 | JF-699 |
| Round 8 | *Tac1* | B1 | AF-488 |
|  | *Cartpt* | B2 | AF-546 |
|  | *Prkcd* | B5 | JF-699 |
| Round 9 | *Sst* | B3 | AF-488 |
|  | *Penk* | B1 | AF-546 |
|  | *Gal* | B5 | JF-699 |
| Round 10 | *Tac2* | B3 | AF-488 |
|  | *Pdyn* | B2 | AF-546 |
|  | *Nts* | B5 | JF-699 |
| Round 11 | *Oprk1* | B1 | AF-488 |
|  | *Scn4b* | B3 | AF-546 |
|  | *Vipr2* | B5 | JF-699 |
| Round 12* | *Scn4b* | B3 | AF-488 |
|  | *Gad1* | B1 | AF-546 |
|  | - | - |  |

*Two genes instead of three genes were probed in round 4, 5 and 12 due to 638 laser failure.
