# Supplementary material for: Multimodal mapping of cell types and projections in the central nucleus of the amygdala
Source: eLife. 2023 Jan 20;12:e84262. doi: 10.7554/eLife.84262 (PMC9977318; doi:10.7554/eLife.84262)
Supplement: Supplementary file 3. [file elife-84262-supp3.docx]

**Supplementary File 3.** Summary of cells analyzed in EASI-FISH

|  |  | All cells | Neurons | Neurons with both molecular and projection info collected |
| --- | --- | --- | --- | --- |
| ANM #1 | A | 6,979 | 5,604 | 5,588 |
|  | M | 5,644 | 4,286 | 3,601 |
|  | P | 6,287 | 4,728 | 2,727 |
| ANM #2 | A | 7,271 | 5,684 | 5,303 |
|  | M | 9,233 | 7,342 | 7,342 |
|  | P | 7,205 | 5,495 | 4,948 |
| Total | | 42,619 | 33,139 | 29,509 |
